# Testing the Reciprocal Effect between Value of Education, Time Investment, and Academic Achievement in a Large Non-Western Sample

**DOI:** 10.3390/jintelligence11070133

**Published:** 2023-07-04

**Authors:** Meimei Liu, TuongVan Vu, Nienke van Atteveldt, Martijn Meeter

**Affiliations:** 1 Department of Educational and Family Studies, Vrije Universiteit Amsterdam, 1081 HV Amsterdam, The Netherlands; 2 Department of Clinical, Neuro- & Developmental Psychology, Vrije Universiteit Amsterdam, 1081 HV Amsterdam, The Netherlands

**Keywords:** value of education, reciprocal effect model, motivation, time investment, academic achievement

## Abstract

Many theories of motivation suggest that motivation and academic achievement reinforce each other over time, yet few longitudinal studies have examined behavioral pathways that may mediate interplay from motivation to achievement. Moreover, empirical studies so far have mostly focused on Western countries. In this study, we first examined whether students’ value of education, as a measure of motivation, is reciprocally related to achievement (class rank and self-rated performance) in a sample of junior high schoolers in an East-Asian country (N = 3445, Korean Youth Panel Study). We tested this reciprocity using different statistical models. Second, we investigated whether the relation between motivation and achievement was mediated by time invested in learning. Reciprocal effects between value of education and academic achievement were found in classic cross-lagged panel models, but only unilateral effects (from achievement to value of education) were found when we used random-intercept and random-curve cross-lagged panel models. Adding the time investment variable, a reciprocal effect between value of education, time investment, and academic achievement was not found with the random-intercept model. In conclusion, the reciprocity between of motivation and achievement are more elusive than previous research suggested; further studies should be dedicated to scrutinizing its existence with various statistical models.

## 1. Introduction

There is no doubt that motivation and achievement are closely related, with the influential reciprocal effects model (REM) theory and many empirical studies suggesting that they are mutually reinforcing (Eccles and Wigfield 2002; Gottfried et al. 2013; Huang 2011; Marsh and Martin 2011; Möller et al. 2011; Niepel et al. 2014; Sewasew and Koester 2019; Valentine et al. 2004; Winne and Nesbit 2010). However, empirical research on interactions between motivation and achievement has not reached full consensus (Burns et al. 2020; Ehm et al. 2019; Hamaker et al. 2015). Moreover, if motivation does influence achievement, little is known about which behavioral pathways mediate between motivation and achievement (Vu et al. 2022). More insight is needed on the causes and effects behind motivation–achievement effects, and in particular the role of mediating behaviors. 

### 1.1. Value of Education

Motivation has been conceptualized in many different ways (Vu et al. 2022). These conceptualizations mostly fall into one of two categories: those related to expectations and self-belief (e.g., self-efficacy, self-concept, locus of control), and those related to the value attached to the activity someone is motivated for (e.g., goal theory, self-determination theory; Eccles and Wigfield 2002; Vu et al. 2022). Previous studies have mostly focused on the first category of motivation variables (Ehm et al. 2019; Marsh et al. 2016; Marsh et al. 2022; Marsh and Martin 2011). For example, a recent meta-analysis (Vu et al., manuscript in revision) on the reciprocal relationship between motivation and academic achievement found that in more than half of the included studies, motivation was operationalized as academic self-concept. There are fewer studies on reciprocity where motivation is operationalized as the value attached to education.

To fill the gap in the literature, the present study focuses on a concept from the second category, the value students attach to attainable goals or aspirations when pursuing education. It has been argued that students who highly value education show more persistence and less drop out (Utvær et al. 2014; Zava et al. 2022). For instance, self-determination theory proposes that life goals or aspirations refer to the long-term aims people value and strive for, and are closely linked to the value they attach to education (Kasser and Ryan 1993, 1996; Utvær et al. 2014). Theoretically, students who attach more value to education are expected to exert more effort and make behavioral choices that lead to higher achievement (Massey et al. 2008).

One well-established measure of general life goals or values is the Aspiration Index (AI) developed on the basis of self-determination theory (Kasser and Ryan 1993, 1996). The index assesses seven dimensions, including wealth, fame, image (physical appearance), personal growth, affiliation, community involvement, and physical fitness. To date, AI has been studied in different cultures and has shown similarities. For example, Schmuck et al. (2000) used AI to examine the structure of college students’ goal content, and found out it was remarkably similar in two cultures (German and US), both of which valued intrinsic goals over extrinsic goals. Similarly, Grouzet et al. (2005) conducted a survey of AI among college students from 15 cultures around the world (including Korea), and they also found that the structure of their intrinsic and extrinsic goals was similar across countries. Therefore, the goals assessed in AI are not limited to Western cultures, but reflect common goals people pursue. The current study focuses only on life goals that are most closely related to education.

### 1.2. Behavior Pathway between Motivation and Achievement

**Figure 1:**
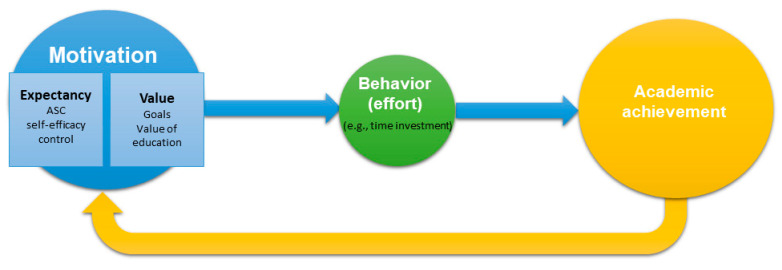
The cyclical relations between motivation and achievement, mediated by effort (adapted from Vu et al. 2022).

Motivation is usually thought to be related to behavior adopted to achieve a certain outcome (Skinner et al. 1990). In fact, many theoretical studies in the educational domain proposed that motivation leads to effortful commitment to learning, which implies that motivation influences achievement through effort (Eccles and Wigfield 2002; Skinner et al. 1990; Vu et al. 2022; see Figure 1). However, this is not always found in empirical studies. Pinxten et al. (2014) first addressed the measurement of effort in a longitudinal study between mathematics self-concept and achievement. They found that math competence beliefs had positive effects on achievement and negative effects on effort. Using the same measures, Marsh et al. (2016) found that the effect of math self-concept on achievement via effort was not significant in most of their models. The difference between theoretical and empirical studies may be due to the fact that there exist different operationalizations and evaluations of effort (Massin 2017; Vu et al. 2022). Specifically, Marsh et al. (2016) and Pinxten et al. (2014) only measured subjective effort, in which students were asked to rate their effort expenditure.

The present study starts from their suggestions that the most feasible pathway from motivation to related achievement is via objective effort put into learning (Marsh et al. 2016; Pinxten et al. 2014). Time invested in learning can be perceived as a quantitative, and thereby more objective, measure of effort as opposed to perceived effort expenditure (Vu et al. 2022). However, the small number of studies that have tested links between time investment and achievement yielded inconsistent findings. When effort is measured as time invested in learning (e.g., time-on-task), the relationship between effort and academic achievement was either negative (Koriat et al. 2006; Nelson and Leonesio 1988; Undorf and Ackerman 2017; Vu et al. 2022) or weakly positive, or only significant after controlling for factors such as learning strategy (Cury et al. 2008; Doumen et al. 2014; Plant et al. 2005). Moreover, to our knowledge, there are no longitudinal studies investigating the reciprocity between motivation and achievement taking into account study time investment. Therefore, this present study examined the mediating role of effort between motivation and achievement longitudinally, using students’ time invested in learning as a measure of effort.

Another gap in the literature is that the reciprocal relationship between motivation and academic achievement has mostly been studied in Western samples. There are at least two reasons why including non-Western samples in motivation–achievement related studies is important. Firstly, a recent meta-analysis by Vu et al. (manuscript in revision, *n* = 47 studies) highlighted a significant underrepresentation of non-Western populations, particularly Asian samples which made up 15% of studies even though 60% of the world’s population resides in Asia. Just one study specifically focused on the Korean population. This limited representation obfuscates whether the reported patterns are universal or in fact culturally specific. Secondly, there are reasons to suspect qualitative differences in the relationship between motivation and achievement across countries with distinct cultural contexts (Vu et al. 2022). For instance, in some cultures that emphasize collectivism, such as East Asian cultures, the pursuit of achievement may be strongly influenced by social harmony and the desire to meet societal expectations. In contrast, individualistic cultures may prioritize personal goals and self-expression as motivators for achievement (Kim 2010). By including diverse samples, this study aims to provide a more comprehensive understanding of whether motivation–achievement relationships are universal, transcending cross-cultural differences.

### 1.3. Testing the Reciprocal Effect Model (REM) Using Different Statistical Models

The relationship between motivation and achievement in most empirical studies was tested so far with the classic cross-lagged panel model (CLPM) (Grygiel et al. 2017; Liu and Hou 2017; Marsh and Martin 2011; Zee et al. 2021). The CLPM has been criticized for confounding between- and within-person processes of change over time (Hamaker et al. 2015). As a consequence, the classic CLPM may not represent the actual within-person growth over time (Ehm et al. 2019). Recent studies focusing on testing different models suggest that modified cross-lagged models that separate these processes from one another such as the random-intercept cross-lagged panel model (RI-CLPM) (Hamaker et al. 2015) and random-curve cross-lagged panel model (RC-CLPM) (Núñez-Regueiro et al. 2021) can remedy the shortcomings of the classic CLPM.

Only a few studies have examined the reciprocity between motivation and academic achievement using such modified models. Their findings suggest that the reciprocity between motivation and academic achievement might be more elusive than previously thought. For example, Ehm et al. (2019) found that the reciprocal effects exist when analyzed with CLPM, but not when using RI-CLPM. Similarly, Burns et al. (2020) re-examined the reciprocal effects model using CLPM and RI-CLPM and they also found reciprocal effects existed when using CLPM, while only the effect of “achievement → motivation” was found with RI-CLPM. More recently, Núñez-Regueiro et al. (2021) further investigated reciprocal effects using both RI-CLPM and RC-CLPM. They also found that while reciprocal effects were found with CLPM, only the effect of “achievement → motivation” was evident with RI-CLPM, while effects in neither direction were present when analyzed with RC-CLPM. Overall, these studies suggest that reciprocal effects are only found using the classical CLPM, and that motivation-to-achievement effect has been sharply curtailed when using models that can distinguish between- and within-person processes of change over time.

## 2. Research Goals and Hypotheses

### 2.1. Present Study

Given the gaps mentioned above, the central aim of the present study was to examine the interplay between value of education (as a measure of motivation), time investment in learning, and how these relate to developments in academic achievement across five years in a non-Western sample of junior high school students. To achieve this, the first step we took was to replicate the findings of previous research that the relationship between motivation and achievement was reciprocal, each construct leading to changes in the other at the subsequent time point. Specifically, we expected to find significant cross-lagged effects between motivation (value of education) and achievement using the classic CLPM. Moreover, we took into account the recent methodological developments and subsequently modeled interactions between value of education and academic achievement with the newer RI-CLPM and RC-CLPM.

The second step was to increase our understanding of mediating behavioral pathways between motivation and achievement. We thus also aimed to investigate the reciprocal effect between motivation, time investment, and achievement. In particular, the RI-CLPM allows us to examine the mediating effect of time investment between motivation and achievement in a trivariate model framework, which is challenging and few studies have performed.

### 2.2. Hypotheses

This study thus has two primary hypotheses. We first expected to find the reciprocal relationship between motivation and academic achievement, replicating previous research when using the CLPM model but not when using the RI-CLPM and RC-CLPM (Hypothesis 1). Second, we expected that motivation is associated with time invested in learning, which in turn is related to achievement, which again is linked with motivation at the next time point (Hypothesis 2).

## 3. Method

### 3.1. Sample

The sample consisted of Korean adolescents who participated in the Korean Youth Panel Survey (KYPS), a panel study conducted by the National Youth Policy Institute (www.nypi.re.kr; accessed on 28 September 2023) to investigate various attitudes of Korea’s youth toward their future, as well as aspects of their academic plans and behaviors. The study focused on the junior high school cohort (KYPS-J). Data from 2003 (second year of junior high school) to 2008 (one year after graduating from high school) for the KYPS-J annual assessment were selected for this current study. The KYPS selected samples through stratified multi-stage cluster sampling where firstly 12 regions were selected, which schools were sampled, and finally randomly selecting one class in each sampled school for surveying. The survey consisted of two components: one for the youth, and another for the parents (guardians). Only data from students were considered in this study.

The KYPS-J consists of 6 waves (2003–2008) from Grade 2 of junior high to Grade 3 of high school, corresponding to International Standard Classification of Education (ISCED) level 2 to level 3. Each wave was collected from the end of October to the end of December. Data from wave 1 to 5 (2003–2007) were extracted for this study, as data from wave 6 were collected after high school graduation and did not have the variables required for this study (including academic performance and time allocated to study). In total, the sample consisted of 3449 students. At each measurement occasion, however, a portion of the sample did not participate. A total of 83.1% of students answered all target questions in wave 1 (*n* = 2865), 77.65% in wave 2 (*n* = 2678), 78.34% in wave 3 (*n* = 2702), 78.78% in wave 4 (*n* = 2717), and 72.43% in wave 5 (*n* = 2498). We excluded four participants that did not respond to any of the variables in all waves. Thus 3445 participants (50% female) were included in the present study. Although Koreans had, by 2023, a different way of calculating age, here we use the globally recognized system of calculating age based on birth date. Using that system, the average age of the first wave in the total sample (*n* = 3445) was slightly under 14 and the last wave was slightly under 18. The data for this secondary analysis, “KYPS, (Korea) National Youth Policy Institute” were provided by the Social Science Japan Data Archive, Center for Social Research and Data Archives, Institute of Social Science, The University of Tokyo. Further information can be found on the website (https://ssjda.iss.u-tokyo.ac.jp/Direct/gaiyo.php?eid=0747).

### 3.2. Measures

#### 3.2.1. Value of Education (Measure of Motivation)

Within the KYPS questionnaires, several questions focused on educational goal orientation, with very similar phrasing as in the Aspiration Index (“how important is this goal to you?”; Kasser and Ryan 1993). We examined three different dimensions of the value of education through four questions, including personal growth (“It is essential to get higher education for self-development”), affiliation (“Getting higher education provides better opportunities for making good friends” and “It is essential to get higher education in order to get an ideal spouse”), and wealth (“It is essential to get higher education in order to get a good job”). Factor analysis was conducted and revealed these four items belonged to the same factor, which we named the value of education, based on its similarity to the Aspiration Index (Kasser and Ryan 1993). A series of confirmatory factor analyses were performed to investigate whether the value of education variables reflected the same constructs across time, resulting in satisfactory findings (see Supplementary Materials Section S1). The answer “I don’t know” was counted as a missing value, all four items were then scored on a 5-point Likert scale, ranging from strongly disagree (1) to strongly agree (5). These were then averaged for each person. Cronbach’s alpha reliability coefficient of the questionnaire in waves 1–5 ranged from 0.82 to 0.84. The full questionnaire can be found on the website (https://ssjda.iss.u-tokyo.ac.jp/Direct/gaiyo.php?eid=0747).

#### 3.2.2. Time Investment

Estimates of time spent on learning were used in the survey as a measure of time investment. Students were asked about how they allocated their time on weekdays and weekends during the semester, as well as during the holidays, to the following five areas: personal care (eating or sleeping), studying in school classes and on their own, studying or tutoring at private academies, part-time work and job preparation, and leisure. The number of hours spent on learning (including school classes, self-study, and private school) was summed to a time investment index. To eliminate time investment outliers, we summed the time allocated to all activities to identify participants who claimed to spend more than 24 h a day. All participants met the criteria and were included in our study.

#### 3.2.3. Academic Achievement

Academic achievement was measured in two ways in the survey: class rank and self-rated performance. Students reported their class rank in the last semester (based on grades for all subjects) as a percentile, ranging from 1% to 100% (1% indicating the best). To avoid negative correlations between achievement and motivation and time investment, we inverted and rescaled the rank achievement variable, ranging from 0 to 1 (1 representing the highest achievement—i.e., 1% rank). Self-rated performance was measured through the question “How well did you do in each subject in your class last term?”. This question was measured for all subjects, using a 5-point Likert scale for all items, ranging from (1) very poor to (5) very good. We used the average score of the three main subjects (National Language—i.e., Korean, Mathematics, and English) as a measure of self-rated performance since these three subjects are mandatory for all students.

#### 3.2.4. Missing Data

The distribution of missing data can be found in Supplementary Materials Table S10. Application of the test suggested by Little and Rubin (2020) showed that the data were not “missing completely at random (MCAR)”, and t-tests showed that missing data had significant differences with non-missing data on every measurement point, and the ANOVA showed that it was also not predicted by any of the variables included in the dataset. This suggests “missing not at random (MNAR)” is the best assumption, meaning that missingness depends on the unobserved data (Tompsett et al. 2018). We therefore employed multiple imputation (ML) by chained equations (MICE) (Azur et al. 2011), a recommended general method for handling missing data, available for most types of data (Jakobsen et al. 2017). The package mice in R was used to address missing data (Van Buuren and Groothuis-Oudshoorn 2011). We first followed the approach suggested by Van Buuren (2012) to create a prediction matrix in a longitudinal dataset, with each variable predicted only by its wave-specific autoregressive and cross-lagged variables and the cross-lagged variables of each wave. Then, 10 imputations were run to obtain robust estimates. Finally, the 95% confidence intervals of estimated autoregressive and cross-lagged parameters for different models were pooled to check for statistical uncertainty. To examine the differences in results between the imputation dataset and the complete cases dataset, we conducted analyses on both datasets and found that the patterns of the results were consistent. The estimated parameters from the complete cases dataset can be found in Supplementary Materials Table S12.

### 3.3. Data Analyses

We first tested the reciprocal effects of motivation (value of education) and achievement through CLPM, RI-CLPM, and RC-CLPM using self-rated performance and rank as outcome variables, respectively. The goal was to better understand the reciprocity of motivation (value of education) and achievement. We expected to find a reciprocal relationship between motivation and academic achievement, replicating previous research when using the CLPM model but not when using the RI-CLPM and RC-CLPM (i.e., Hypothesis 1). To further examine the mediating role of time investment between value of education and achievement, we constructed a trivariate RI-CLPM with time investment as the mediator and self-rated performance and rank as outcome variables separately (i.e., Hypothesis 2). We did not construct trivariate RC-CLPM due to the fact that this model required a sizeable number of covariance parameters which made the model too complex.

All analyses were performed with R using the lavaan package and based on the R scripts by Usami et al. (2019) and Núñez-Regueiro et al. (2021). The full R script in the present study can be found in Supplementary Materials Section S3. All models were subjected to equality constraints on autoregressive and cross-lagged effects, meaning that autoregressive and cross-lagged effects were set equal in each wave. The modelling results are based on multiple imputations of missing values. The 10 imputations were used to compute 95% confidence intervals for each estimated parameter for the autoregressive and cross-lagged effects in the different models. For the fit indices and significance effects, we report results from the optimal imputation selected as the one with lowest AIC and BIC.

We compared models via the chi-square test, and applied both the absolute and incremental fit indexes to evaluate the overall model fit, which includes the root mean square error of approximation (RMSEA; acceptable fit: <.08, good fit: <.05) (Steiger 1990), the comparative fit index (CFI; acceptable fit: .95–.97, good fit; >.97) (Hu and Bentler 1998), the Tucker–Lewis Index (TLI; acceptable fit: >.90, good fit; >.95) (Bentler and Bonett 1980), the standardized root mean squared residual (SRMR; acceptable fit: <.10, good fit; <.05), and information criteria such as Akaike’s information criteria (AIC) (Hu and Bentler 1998; Xia and Yang 2019). We report these fit indices and the standardized coefficients of models to ease parameter comparisons as they are the most representative indices to estimate model fit (Xia and Yang 2019).

## 4. Result

The results are divided into four sections. First, we report descriptive statistics and correlations for motivation (value of education), time investment, and the two achievement measures (self-rated performance and class rank). Secondly, we present the results of three models (CLPM, RI-CLPM, RC-CLPM) analyzing the longitudinal association between value of education and academic achievement (separately for class rank and self-rated performance). Finally, we present the results of a longitudinal link between value of education, time investment, and achievement when using the RI-CLPM (also separate for the two achievement indicators).

### 4.1. Descriptive Statistics

Table 1 shows descriptive statistics and correlations for the self-rated performance of three subjects, rank in class, value of education, and time investment in learning. The correlations showed a medium to high correspondence in rank from one point in time to the next (ranging from .41 to .83) and time invested in learning (ranging from .43 to .68). The stability of the value of education increased from r = .41 (between grade 2 and 3 in junior high school) to r = .52 (between grade 2 and 3 in high school). From grade 2 of junior high school and grade 3 of high school, the average number of hours invested in learning over a two-week period increased from 27.60 to 40.76 and students’ value of education increased from 3.08 to 3.35.

### 4.2. Cross-Lagged Panel Model

**Figure 2:**
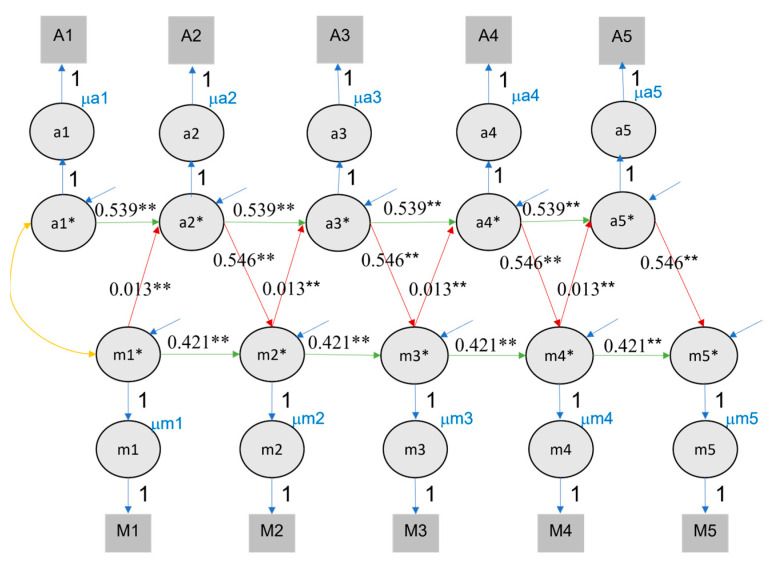
CLPM fits of longitudinal interactions between value of education and class rank. Note. M/m: motivation ratings, defined as value of education, A/a: achievement measure of class rank. Square and capital letters refer to observed variables, circles and small letters to latent ones. Value of education and achievement were both queried in November/December, but the reported achievement referred back to academic performance in the previous semester, usually between March and June. To reflect this, value of education lags achievement in the figure. The numbers labelling red and green lines indicate the midpoints of the 95% confidence intervals for cross-lagged effect and autoregressive effects, respectively. ** indicates that the *p* value of significant effects is lower than 0.01, * indicates that the *p* value of significant effects is lower than 0.05.

**Figure 3:**
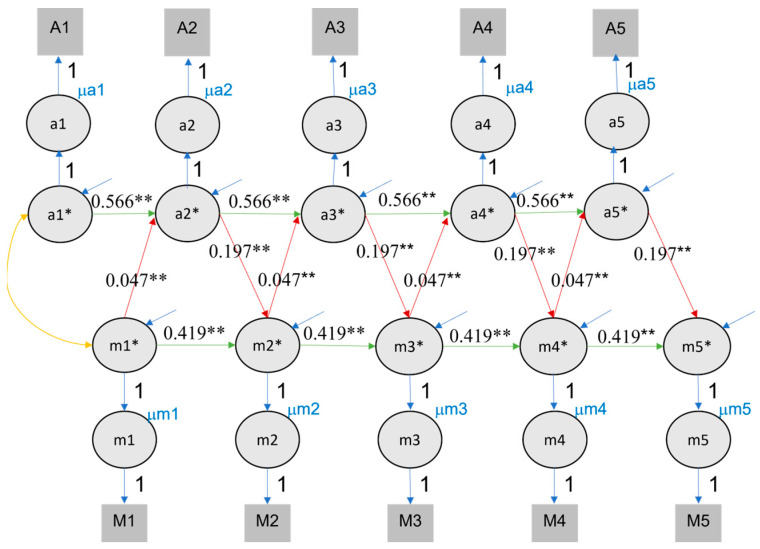
CLPM fits of longitudinal interactions between value of education and self-rated performance. Note. Conventions as in Figure 2.

A comparison of the AIC and BIC for the ten times imputations using an ANOVA revealed that the fourth imputation for models fitted on the data including self-rated performance data and the sixth model for the data including class rank was optimal, as their AIC and BIC indicate that they are the best-fitting models (see Supplementary Materials Section S2), thus we report their fit indices. Even though the CLPM showed a mediocre fit for both self-rated performance (CFI = .840, TLI = .844, RMSEA = .110, SRMR = .106) and class rank (CFI = .812, TLI = .816, RMSEA = .116, SRMR = .106), we still reported them for model comparison. Significant cross-lagged effects were found for value of education on rank (ranging from .013 to .015) and rank on value of education (ranging from .532 to .544) from one time point to the next (see Figure 2). Conversely, effects were also found for value of education on self-rated performance (ranging from .044 to .047) and self-rated performance on value of education (ranging from .196 to .200) from one time point to the next (see Figure 3).

### 4.3. Random-Intercept Cross-Lagged Panel Model

**Figure 4:**
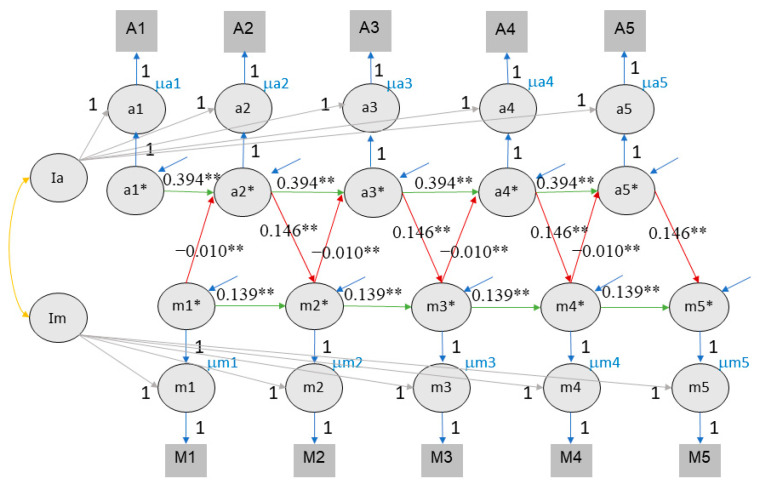
RI-CLPM fits for value of education and class rank. Note. Im indicates the intercept of motivation (value of education), Ia indicates the intercept of achievement. For visual clarity, variances, and covariance, latent structure for e* are not included in the picture but are included in the analysis. ** indicates that the *p* value of significant effects is lower than 0.01, * indicates that the *p* value of significant effects is lower than 0.05.

**Figure 5:**
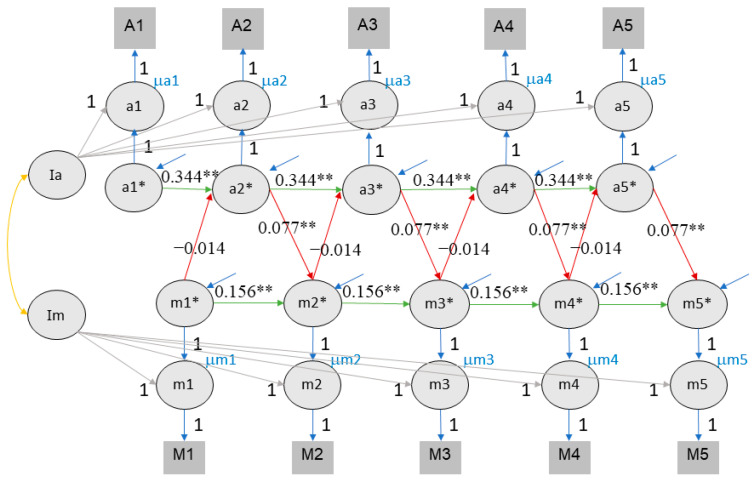
RI-CLPM fits for value of education and self-rated performance. Note. Conventions as in Figure 4.

The RI-CLPM had a better fit than the CLPM when estimating the effects of value of education and self-rated performance (CFI = .922, TLI = .918, RMSEA = .080, SRMR = .068) and value of education and rank (CFI = .888, TLI = .883, RMSEA = .092, SRMR = .071). In contrast to the CLPM results, no reciprocal effects were found using this model (see Table 2 and Figure 4 and Figure 5). Unilateral effects of achievement on value of education were found for both class rank (ranging from .138 to .159) and self-rated performance (ranging from .075 to .083). The effect of value of education on achievement was negative in terms of rank, ranging from −.010 to −.008 (note that our measure of rank was reversed and thus a positive effect was expected), and was not significant in relation to self-rated performance (see Figure 4 and Figure 5).

### 4.4. Random-Curve Cross-Lagged Panel Model

**Figure 6:**
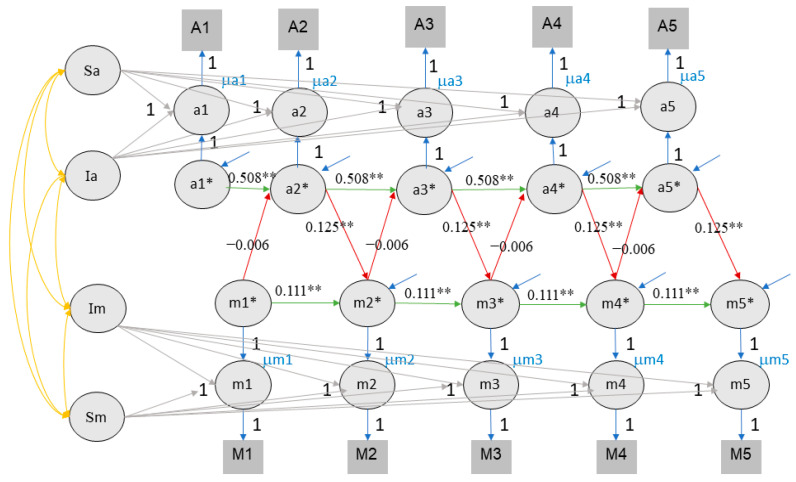
The RC-CLPM of value of education and class rank. Note. Sa stands for the slope of achievement, Sm stands for the slope of value of education. ** indicates that the *p* value of significant effects is lower than 0.01, * indicates that the *p* value of significant effects is lower than 0.05.

**Figure 7:**
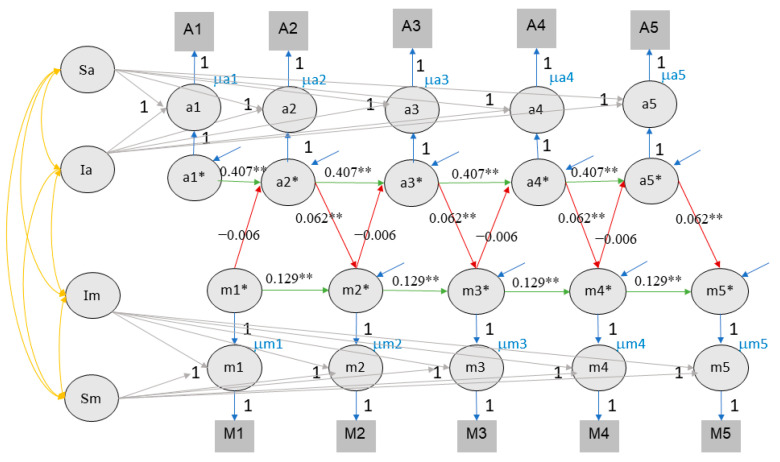
The RC-CLPM of value of education and self-rated performance. Note. Conventions as in Figure 6.

When the data were fitted with the RC-CLPM, the model showed an acceptable fit for both class rank (CFI = .932, TLI = .923, RMSEA = .075, SRMR = .074) and self-rated performance scores (CFI = .929, TLI = .920, RMSEA = .077, SRMR = .079). With the inclusion of slope, similar to the results of the RI-CLPM, there was again no reciprocal relationship between value of education and academic achievement, for both the rank and self-rated performance measures. Only a significant unilateral effect of achievement on value of education was found for both class rank (95% CI of ten times imputation ranging from .118 to .139) and self-rated performance (95% CI ranging from .058 to .067). However, the effect of value of education on achievement was not significant for both achievement measures (see Figure 6 and Figure 7).

As shown in Table 2, the RI-CLPM and RC-CLPM showed a better fit than the CLPM, and they both supported the same pattern of relations between value of education and achievement: no reciprocal effect, but only a positive effect from achievement to value of education.

### 4.5. Longitudinal Association of Value of Education, Time Investment and Academic Achievement

**Figure 8:**
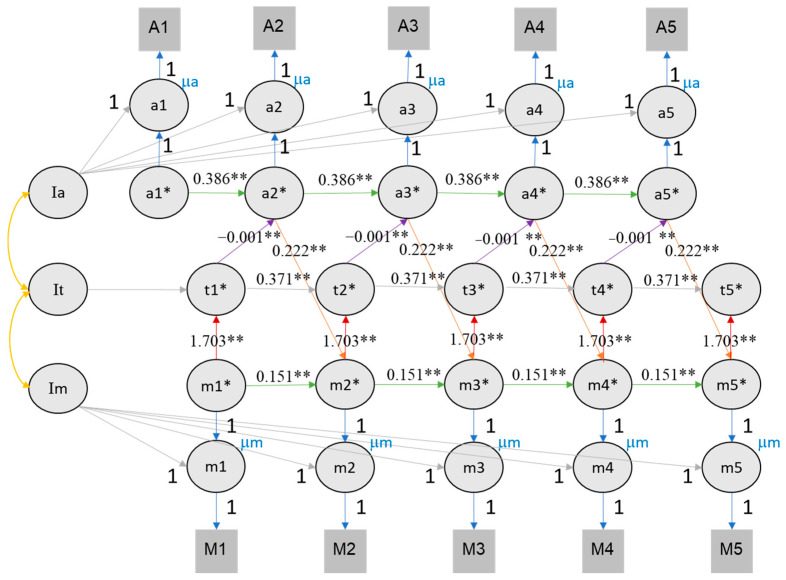
The RI-CLPM of value of education, time investment, and class rank. Note. Achievement here refers to self-rated performance in three subjects during the last semester. It is assumed that the achievement in the previous semester influences the motivation in the subsequent semester, which in turn affects the time investment in the current semester. The figures illustrate the reciprocal relationships among motivation (M/m), achievement (A/a), and time investment (t). Im, It, and Ia represent the intercepts of motivation, time investment, and achievement, respectively. Lowercase letters denote the time points from wave 1 to 5. Circles represent latent variables, while rectangles represent observed variables. The cross-lagged parameters are color coded (red, purple, and orange) to indicate different relationships, and autoregressive parameters are shown in green. The autoregressive and cross-lagged parameters are all equally constrained, hence the equal number of lines representing each parameter. The figures exclude the latent structure for error terms (e*) and associated variances and covariances for visual clarity. ** indicates that the *p* value of significant effects is lower than 0.01, * indicates that the *p* value of significant effects is lower than 0.05.

**Figure 9:**
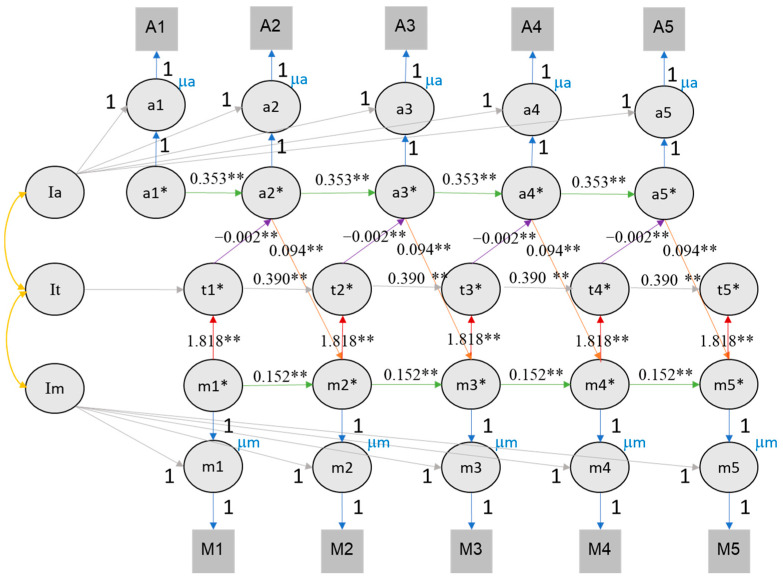
The RI-CLPM of value of education, time investment, and self-rated performance. Note. Conventions as in Figure 8. ** indicates that the *p* value of significant effects is lower than 0.01, * indicates that the *p* value of significant effects is lower than 0.05.

To evaluate the role of time investment as mediating the effect between value of education and achievement, we expanded the RI-CLPM by including the time investment variable (see Figure 8 and Figure 9). For this analysis, we only considered RI-CLPM and not RC-CLPM to limit complexity (given the many covariance parameters introduced in a trivariate RC-CLPM). Table 3 provides a comprehensive overview of all trivariate model parameters. The trivariate RI-CLPM model showed an acceptable fit for both self-rated performance (CFI = 0.842, TLI = 0.833, RMSEA = 0.091, SRMR = 0.128) and class rank (CFI = 0.809, TLI = 0.797, RMSEA = 0.099, SRMR = 0.135). In the trivariate RI-CLPM, cross-lagged effects are represented by three links: value of education → time investment, time investment → achievement, achievement → value of education. Following the recommendations of Núñez-Regueiro et al. (2021), we report fit indices and significant effects for the fourth imputation for both rank and self-rated performance (see Supplementary Materials Section S2).

For class rank, a cross-lagged effect between value of education, time investment, and rank was found. The effect of value of education on time investment ranged from 1.729 to 2.137, the effect of time investment on rank ranged from −0.001 to 0.000, and that of rank on value of education ranged from 0.176 to 0.258 (see Figure 8). It is important to consider that the magnitude of these coefficients is influenced by the larger scale of time investment compared to other measures. 

For self-rated performance, we found the same reciprocal effects between value of education, time investment, and achievement. From each time point to the next, the 95% confidence intervals of value of education to time investment effect ranged from 1.914 to 2.220, the effect of time investment on self-rated performance ranged from −0.001 to 0.000 (again affected by the larger scale of time investment), and the effect of self-rated performance scores on value of education ranged from 0.092 to 0.112 (see Figure 9).

## 5. Discussion

This study aimed to explore the mediating role of time investment between motivation and achievement, and investigate reciprocal interactions between motivation and achievement using modified cross-lagged models. Additionally, we aimed to extend previous research by examining value of education as a motivation concept and including a non-Western sample.

We first examined the reciprocal relationship between value of education and achievement. When fitting CLPM, RI-CLPM, and RC-CLPM to continuous five-year high school data from South Korea, reciprocal effects between value of education and achievement were only found in CLPM. No such effects were found for RI-CLPM and RC-CLPM; in these two models, only the positive effect of achievement on value of education was significant, not the reverse pathway (which was even slightly negative for RI-CLPM and class rank). This indicates that whether reciprocal effects between value of education (as motivation measure) and achievement are found, is a function of the statistical modelling technique used. Then, we expanded the RI-CLPM by including the student’s time investment to check whether time investment mediates between value of education and achievement. We found that there is a reciprocal relationship between value of education, time investment, and academic performance. We discuss each of the findings from the methodological and theoretical perspectives below.

### 5.1. Reciprocal Interactions between Motivation and Achievement: Model Dependent

Cross-lagged associations between motivation and achievement were found using CLPM. This is consistent with previous studies demonstrating reciprocal effects between motivational constructs and achievement (Grygiel et al. 2017; Liu and Hou 2017; Marsh and Martin 2011; Schiefele et al. 2016; Zee et al. 2021). However, the CLPM has been criticized for the confusion of within-person processes and stable between-person differences, implying that the cross-lagged regressions in the CLPM may not represent actual within-person relationships over time (Hamaker et al. 2015). Therefore, Hamaker et al. (2015) and Núñez-Regueiro et al. (2021) proposed the inclusion of random-intercepts (i.e., the RI-CLPM) or random-intercepts and slopes (i.e., the RC-CLPM) into CLPM. Our findings with RI-CLPM and RC-CLPM suggest that there are no reciprocal interactions between value of education and achievement, there were only the unilateral positive effects from achievement to value of education. These results dovetail with those of some prior studies. Ehm et al. (2019) compared the reciprocal effects of motivation, measured as self-concept, and achievement in five models (including CLPM and RI-CLPM). The conclusion they arrived at is that different reciprocal effect models lead to different results. Burns et al. (2020) examined the reciprocal effects between self-concept and academic achievement in RI-CLPM. They also found that only the link from achievement to motivation was evident. Núñez-Regueiro et al. (2021) similarly found only an effect of achievement on motivation with RI-CLPM, while effects in neither direction are present when analyzed with RC-CLPM. Thus, our Hypothesis 1 did not receive support, as different models led to different conclusions regarding the reciprocal relationship between value of education and achievement when using different models.

The reason different models can lead to different results is rooted in their underlying specifications of individual differences. The CLPM assumes that trajectories over time are independent of individual differences. This has been criticized because, mathematically, stable individual differences tend to leak into estimates of within-person processes (Núñez-Regueiro et al. 2021). In contrast, the RI-CLPM and RC-CLPM model stablize individual differences by introducing random-intercepts and slopes. By incorporating stable individual variation, these models can provide a more accurate representation of within-person dynamics. Consequently, the estimated effects and patterns observed in the RI-CLPM and RC-CLPM may differ from the CLPM. This means that cross-lagged effects deemed significant in the CLPM may be rendered nonsignificant in the RI-CLPM and RC-CLPM, and vice versa.

Given the dependence of conclusions on the used statistical model, the question becomes, which model best describes the relations between value of education and achievement? Fit indices are instructive for finding an optimal model (Usami et al. 2019). In line with prior research (Núñez-Regueiro et al. 2021), the present study found a better fit of the data for the RI-CLPM and RC-CLPM than for the CLPM. For Núñez-Regueiro and colleagues, their results were reason to question the causal framework of motivation and achievement, since the evidence is vulnerable to statistical artifacts which, they argue, are less severe with newer modelling strategies (i.e., RI-CLPM and RC-CLPM). With the appearance of more advanced models, whether reciprocal effects between motivation and achievement will still be found in future meta-analyses is a matter of concern.

### 5.2. Time Investment as a Mediating Factor in the Effect between Motivation and Achievement

It is vital to understand the mechanism of mediating pathways from motivation to achievement (Vu et al. 2022). To the best of our knowledge, our study is the first in which time investment is included as a mediator between motivation and achievement in a longitudinal study within a non-Western high school context. A possible explanation is the challenge to construct a trivariate cross-lagged effect model. So far, only one academic motivation paper constructed a trivariate CLPM and RI-CLPM, in their case between self-concept, grade, and standardized test scores (Marsh et al. 2022). The rationale of our trivariate RI-CLPM was based on Figure 1. We hypothesized that motivation was positively related to their present time investment, and that time investment was positively related to subsequent achievement, which in turn correlated with subsequent motivation. Our results for the trivariate RI-CLPM showed this and supported our Hypothesis 2, that there is a reciprocal effect between value of education, time investment, and academic achievement. We discuss the three links (motivation → time investment, time investment → achievement, and achievement → motivation) and then our achievement and motivation indicators below.

Motivation → time investment: Value of education was positively related to time investment in our study. This result supports our assumption (Hypothesis 2). Theoretically, according to expectancy-value theory, students’ willingness to invest time and effort in a task can be explained by task-value beliefs, that is, beliefs about the importance, interest, and value of the task (Eccles and Wigfield 2002). This suggests a positive correlation between task value beliefs (e.g., value of education) and effort, which is consistent with our results. Our finding is also in line with Timmers et al. (2013). They investigated time invested in a specific task as a measure of effort, and found that this was positively related to task-value beliefs and not with expectancy of success.

Time investment → achievement: Time investment was negatively, but weakly, correlated with achievement in the rank and self-assessed performance measures. This suggests that increased time investment does not lead to improved outcomes; in fact, it is negatively correlated with academic achievement. Similarly, (Marsh et al. 2016) examined the longitudinal reciprocal effects of academic self-concept, effort, and academic achievement among thousands of adolescents, and found that prior effort had non-significant or negative effects on subsequent grades. These results suggest that investing more time in learning may not always lead to higher achievement.

Achievement → motivation: Our two achievement indicators were both positively associated with value of education. The effect of class rank on value of education was almost twice as large as the effect of self-rated performance on value of education. These findings were consistent with our results of three bivariate models. Overall, our study suggested a positive link from achievement to motivation, replicating many previous studies (Burns et al. 2020; Vu et al., manuscript in revision). For example, Burns et al. (2020) examined the reciprocal effect between self-concept and academic achievement; they found that using RI-CLPM achievement affected self-concept, not vice versa.

Rank and self-rated performance: Even though our two achievement indicators consistently yielded the same results, one may still wonder which achievement indicator is more representative. The merits of different ways of assessing achievement are an ongoing concern in REM research. Marsh and Martin (2011) believed school grades, such as class rank, provide a more salient and local source of feedback to students about their accomplishments compared to standardized test scores, and thus tend to be more correlated with motivation. Furthermore, Marsh et al. (2014) proposed that high-stakes tests might result in stronger links between achievement, motivation, and effort than low-stakes tests. If the tests are critically important for students, they may be highly motivated to prepare for the test, and thus, effort may then be more positively related to motivation. Some studies making use of different achievement measures found inconsistent results (Marsh et al. 2016; Marsh et al. 2022). For instance, Marsh et al. (2016), using two achievement measures (grades and test scores), found a nonsignificant effect of academic self-concept and effort on grades, but positive ones on test scores.

For our two measures, rank and self-rated performance measures, there are two main differences. First, self-rated performance was averaged in three subjects while rank is based on five subjects (social science and science are the other two subjects). When we correlated the average self-rated performance for the three core subjects with that of all five subjects taken by students in each wave, the two averages were highly correlated, ranging from 0.83 to 0.93 (see Supplementary Materials Table S11). This suggests that the difference in subjects is not a salient factor for our results. Second, there may be an element of subjectivity in the self-ratings, and to some extent a student’s self-rating may already measure motivation. However, the correlation between self-rated performance and motivation (ranging from 0.21 to 0.33) is not higher than class rank and motivation (0.19 to 0.32). Self-rated performance may therefore not already measure motivation to a larger extent than class rank. Indeed, the results for our two achievement indicators were equal at a qualitative level.

Motivation measure: A difference between our study and most REM studies is that they focus on motivational variables related to self-belief (e.g., self-efficacy or self-concept) (Burns et al. 2020; Ehm et al. 2019; Marsh et al. 2022; Marsh and Martin 2011), rather than value of education. Our study is the first to focus on motivation as the value attached to education. Motivation theories postulate that students who attach more value to education should exert more effort for higher achievement, which would then lead to stronger value being attached to education (Eccles and Wigfield 2002; Skinner et al. 1990; Vu et al. 2022). Indeed, we detected such reciprocity in the trivariate RI-CLPM. However, we did not find it in the corresponding bivariate model, where the effect of value of education on achievement was not significant using RI-CLPM. In traditional approaches to mediation analysis, the direct effect of motivation to achievement was a prerequisite for a mediating effect. However, this has been long criticized by methodologists who argue that significant indirect effects may point to mediation even if there are nonsignificant direct effects (Rucker et al. 2011). Therefore, our trivariate finding of a mediated pathway from motivation, through time investment, to achievement, may be taken at face value. An explanation for the discrepancy between the bivariate and trivariate model results could be that this mediated effect might be too small or being suppressed by an unknown variable to lead to a significant, unmediated path from motivation to achievement.

### 5.3. Non-Western Sample in REM Study

The present study is the first to investigate the reciprocal effect between value of education and achievement in non-Western samples. The latest meta-analysis (Vu et al., manuscript in revision) suggests that the country of origin could be one of moderators between relations of motivation and achievement. Several aspects are noteworthy to discuss when comparing Western and non-Western samples. First, in both non-Western and Western samples reciprocal effect between motivation and achievement are typically found when using CLPM (Grygiel et al. 2017; Liu and Hou 2017; Marsh and Martin 2011; Schiefele et al. 2016; Zee et al. 2021). Second, our finding that the reciprocity is found with CLPM but not newer models is consistent with three prior studies involving Western samples (Burns et al. 2020; Ehm et al. 2019; Núñez-Regueiro et al. 2021). The commonalities we found may mean that these findings describe the broad psychological nature of the interplay between motivation and achievement, which is not highly susceptible to cultural context.

## 6. Conclusions

Many studies have shown that motivation and achievement influence each other over time (Marsh and Martin 2011). However, these relations have rarely been investigated using modified cross-lagged models that include random-intercepts and slopes, and the related studies are heavily skewed to Western samples. Moreover, few studies have examined the mediating effect of effort (time investment) between motivation and achievement in the longitudinal framework. In the present study, using a more appropriate modelling strategy and non-Western high school samples, we found that the reciprocal effect between value of education and academic achievement is only present using the classical CLPM (which fitted the data only weakly). When fitting the data with the RI-CLPM and RC-CLPM, which take between- and within-person variation into account, only the unidirectional effect of achievement on value of education is evident. We thus found that whether reciprocal effects exist depends on the statistical models we used. In the trivariate RI-CLPM, reciprocal effects from value of education to time investment, to academic achievement and back to value of education were found. In conclusion, the reciprocity of motivation and achievement is more elusive than previous research suggests. Further studies should scrutinize its existence with various statistical models other than the CLPM.

## 7. Limitations and Future Research

A limitation of this study was that it relied mostly on self-report measures of variables, including academic achievement. Although self-report measures are susceptible to biases (e.g., self-serving biases), the intrinsic nature of motivational beliefs renders self-report one of the most appropriate measures (Stone 2000). However, for achievement, recorded grades would be preferable because of the risk of either memory failure or bias. Furthermore, it is important to note that in the present study, the measurement of effort as a behavioral pathway from motivation to achievement only considered time investment. Based on the review by Vu et al. (2022), additional pathways between motivation and achievement could include factors such as persistence (Cury et al. 2008; Doumen et al. 2014), and the utilization of learning strategies (self-determination theory, Ryan and Deci 2000). Moreover, pathways back from achievement to motivation could involve perceived achievement (expectancy value theory, Eccles and Wigfield 2002) or the experience of flow (flow theory, Csikszentmihalyi 1990). Therefore, further investigation into additional mediators on the pathways between motivation and achievement is warranted.

Another limitation of the present study is that it adopted a trivariate RI-CLPM rather than RC-CLPM. With the bivariate models, we showed that the reciprocity between motivation and achievement depends on the statistical model used, and whether the reciprocity between motivation, time investment, and achievement also depends on models is thus a concern. Due to the complexity of model construction, this study did not conduct the extended trivariate RC-CLPM, a model that fits better than the RI-CLPM in a bivariate model. It would be beneficial for future research to examine the reciprocal effects in a trivariate RC-CLPM framework. Except for those models mentioned above, recent methodological approaches such as Bayesian estimation for time series and panel data models can be explored, offering a promising avenue for future research. Bayesian estimation allows for the incorporation of prior information and the estimation of uncertainty, potentially providing researchers with new insights and a more comprehensive understanding of the underlying dynamics and relationships within the data.

Finally, it is worth noting that the dataset utilized in the current study dates back 20 years, highlighting the need to acknowledge that the extent of technology usage among adolescents was significantly lower compared to the present day. However, it is crucial to recognize that technology can influence both the value of education and the time spent on schoolwork. Future research should consider the impact of technology on learning as an additional factor to explore. This will provide valuable insights into how technology shapes students’ motivation and academic outcomes in modern contexts.

## Figures and Tables

**Table 1:** Descriptive statistics and correlations of the value of education, time investment, rank, and self-rating performance of three subjects.

	M	sd	m1	m2	m3	m4	m5	s1	s2	s3	s4	s5	t1	t2	t3	t4	t5	r1	r2	r3	r4
m1	3.08	0.87	1																		
m2	3.16	0.91	.41 **	1																	
m3	3.24	0.88	.32 **	.46 **	1																
m4	3.36	0.86	.29 **	.44 **	.52 **	1															
m5	3.35	0.87	.28 **	.40 **	.43 **	.51 **	1														
s1	3.13	0.82	.25 **	.30 **	.31 **	.29 **	.28 **	1													
s2	3.12	0.84	.24 **	.33 **	.31 **	.29 **	.29 **	.74 **	1												
s3	3.20	0.73	.14 **	.16 **	.23 **	.19 **	.19 **	.45 **	.46 **	1											
s4	2.98	0.71	.11 **	.17 **	.19 **	.21 **	.23 **	.40 **	.40 **	.60 **	1										
s5	3.02	0.70	.12 **	.17 **	.20 **	.22 **	.26 **	.43 **	.44 **	.56 **	.65 **	1									
t1	27.60	13.08	.18 **	.17 **	.17 **	.16 **	.15 **	.29 **	.25 **	.19 **	.18 **	.19 **	1								
t2	27.06	13.43	.21 **	.24 **	.22 **	.22 **	.22 **	.34 **	.37 **	.23 **	.21 **	.21 **	.43 **	1							
t3	34.48	17.68	.21 **	.27 **	.31 **	.29 **	.26 **	.48 **	.49 **	.31 **	.28 **	.31 **	.38 **	.49 **	1						
t4	38.38	20.09	.21 **	.28 **	.30 **	.34 **	.30 **	.50 **	.49 **	.30 **	.30 **	.32 **	.36 **	.45 **	.68 **	1					
t5	40.76	25.11	.15 **	.24 **	.27 **	.30 **	.30 **	.41 **	.40 **	.22 **	.22 **	.31 **	.30 **	.36 **	.51 **	.58 **	1				
r1	44.98	27.73	.24 **	.32 **	.30 **	.30 **	.29 **	.72 **	.68 **	.37 **	.35 **	.38 **	.23 **	.33 **	.49 **	.54 **	.47 **	1			
r2	41.36	26.90	.23 **	.32 **	.33 **	.33 **	.31 **	.69 **	.70 **	.38 **	.37 **	.41 **	.22 **	.36 **	.53 **	.56 **	.50 **	.83 **	1		
r3	39.64	25.19	.11 **	.15 **	.20 **	.21 **	.20 **	.35 **	.34 **	.62 **	.53 **	.51 **	.13 **	.18 **	.27 **	.27 **	.20 **	.39 **	.41 **	1	
r4	40.81	24.41	.09 **	.11 **	.17 **	.19 **	.20 **	.29 **	.30 **	.49 **	.62 **	.54 **	.12 **	.16 **	.24 **	.27 **	.21 **	.34 **	.36 **	.67 **	1
r5	39.68	23.72	.08 **	.11 **	.18 **	.19 **	.22 **	.32 **	.30 **	.50 **	.56 **	.65 **	.12 **	.15 **	.24 **	.27 **	.25 **	.36 **	.39 **	.64 **	.73 **

Note. ** *p* < 0.01 level (2-tailed), m1–m5: value of education in wave 1–5, s1–s5: self-rating performance in wave 1–5, t1–t5: time investment (in hours in two weeks, on top of school time) in learning in wave 1–5, r1–r5: rank in wave 1–5 (note with rank here refers to reversed rank in wave 1–5, higher rank value represent better academic achievement, hence no negative correlations are observed).

**Table 2:** Model comparison of the bivariate models of value of education and achievement (self-rated performance and class rank).

	Goodness-of-Fit Indices					
	*χ*^2^(*df*)	CFI	RMSEA	TLI	SRMR	AIC
(A) CLPM						
(1) Value of education and self-rated performance	1749.43(46)	.840	.110	.844	.106	73,306.71
(2) Value of education and class rank	1947.71(46)	.812	.116	.816	.106	36,993.92
(B) RI-CLPM						
(1) Value of education and self-rated performance	869.20(43)	.922	.080	.918	.068	72,330.67
(2) Value of education and class rank	1166.68(43)	.888	.092	.883	.071	36,135.89
(C) RC-CLPM						
(1) Value of education and self-rated performance	717.70(40)	.938	.073	.931	.067	72,133.92
(2) Value of education and class rank	746.89(40)	.932	.075	.923	.074	35,645.36

Note. CLPM (cross-lagged panel model), RI-CLPM (random-intercept cross-lagged panel model), RC-CLPM (random-curve cross-lagged model). CFI refers to the robust comparative fit index. TLI refers to robust Tucker–Lewis Index, RMSER refers to robust root mean square error of approximation.

**Table 3:** Parameters estimates from different models.

Parameters	CLPM		RI-CLPM		RC-CLPM		3 Variable RI-CLPM		
Autoregressive Effects (β)	Value of Education	Achievement	Value of Education	Achievement	Value of Education	Achievement	Value of Education	Time Investment	Achievement
(1) value of education and self-rated performance	0.419 ***	0.566 ***	0.156 ***	0.344 ***	0.129 ***	0.407 ***			
(2) value of education and class rank	0.424 ***	0.541 ***	0.137 ***	0.395 ***	0.115 ***	0.506 ***			
(3) value of education, time investment, and self-rated performance							0.152 ***	0.390 ***	0.353 ***
(4) value of education, time investment, and class rank							0.151 ***	0.371 ***	0.386 ***
Cross-lagged effects (γ)	Value of education → achievement	Achievement → Value of education	Value of education → achievement	Achievement → Value of education	Value of education → achievement	Achievement → Value of education	Value of education → time investment	Time investment → achievement	Achievement → value of education
(1) value of education and self-rated performance	0.047 ***	0.197 ***	−0.014	0.077 ***	−0.006	0.062 ***			
(2) value of education and class rank	0.013 ***	0.546 ***	−0.010 ***	0.146 ***	−0.006	0.125 ***			
(3) value of education, time investment, and self-rated performance							1.818 ***	−0.002 ***	0.094 ***
(4) value of education, time investment, and class rank							1.703 ***	−0.001 ***	0.222 ***
Random effects and covariances	Value of education and achievement		Value of education and achievement		Value of education and achievement		Value of education and time investment	Time investment and achievement	Achievement and value of education
Intercept									
self-rated performance			0.141 ***		0.184 ***		3.569 ***	2.716 ***	0.137 ***
class rank			0.046 ***		0.065 ***		2.819 ***	1.187 ***	0.043 ***
Slope									
self-rated performance					0.002 ***				
class rank					0.001 **				
Intercept and slope									
self-rated performance					−0.023 ***				
class rank					−0.010 **				

Note: *** indicates that the *p* value of significant effects is lower than 0.001, ** indicates that the *p* value of significant effects is lower than 0.01.

## Data Availability

Data used in this study are the proprietary property of the Social Science Japan Data Archive (SSJDA). All data are solely owned and licensed by the Social Science Japan Data Archive and thus cannot be shared by the authors in any form or format. Requests to access the data should be directed to the Social Science Japan Data Archive.

## Supplementary Materials

The following supporting information can be downloaded at: https://www.mdpi.com/article/10.3390/jintelligence11070133/s1. Testing the reciprocal effect between value of education, time investment, and academic achievement in a large non-Western sample|Zenodo; Section S1: Factor analysis of motivational variables and missing data; Table S1: Codes of motivational questionnaire; Figure S1: The factor analysis of 17 motivational items in wave 1; Figure S2: The factor analysis of 17 motivational items in wave 2; Figure S3: The factor analysis of 17 motivational items in wave 3; Figure S4: The factor analysis of 17 motivational items in wave 4; Figure S5: The factor analysis of 17 motivational items in wave 5; Section S2: Multiple imputation results; Table S2: 10 times imputation on CLPM in rank; Table S3: 10 times imputation on RI-CLPM in rank measures; Table S4: 10 times imputation on RC-CLPM in rank measures; Table S5: 10 times imputation on trivariate RI-CLPM in rank measures; Table S6: 10 times imputation on CLPM in self-rated performance measures; Table S7: 10 times imputation on RI-CLPM in self-rated performance measures; Table S8: 10 times imputation on RC-CLPM in self-rated performance measures; Table S9: 10 times imputation on trivariate RI-CLPM in self-rated performance measures; Table S10. The distribution of missing data; Table S11: The correlation between the average self-rated scores of five subjects, three subjects, and class rank; Table S12: Parameter estimates from different models using data with complete cases. Section S3: codes of modelling.
