# Correction: Liu et al. (2023). Testing the Reciprocal Effect between Value of Education, Time Investment, and Academic Achievement in a Large Non-Western Sample. *Journal of Intelligence* 11: 1333

**DOI:** 10.3390/jintelligence12110117

**Published:** 2024-11-14

**Authors:** Meimei Liu, TuongVan Vu, Nienke van Atteveldt, Martijn Meeter

**Affiliations:** 1 Department of Educational and Family Studies, Vrije Universiteit Amsterdam, 1081 HV Amsterdam, The Netherlands; 2 Department of Clinical, Neuro- & Developmental Psychology, Vrije Universiteit Amsterdam, 1081 HV Amsterdam, The Netherlands

In a recent paper, while our overall conclusion remains valid, one specific finding—that there are reciprocal relationships between how Korean students value education, the time they invest in their studies, and their academic achievements—requires correction (Liu et al. 2023). Unfortunately, this conclusion was partly based on erroneous model specifications. The tri-variate models that were used for hypothesis testing were incorrectly constructed due to the omission of a fixed intercept and the variance of time investment. This led to erroneous results reported in Table 3 and Figures 8 and 9 of the original publication. After correction, we found no positive effect of time investment on achievement, but only unidirectional effects of value of education on time investment and of achievement on value. The corrected Table 3 is provided below, with the changes that led to the controversial conclusion highlighted in bold and underlined:

**Table 3:** Parameters estimates from different models.

Parameters	CLPM		RI-CLPM		RC-CLPM		3 Variable RI-CLPM		
Autoregressive Effects (β)	Value of Education	Achievement	Value of Education	Achievement	Value of Education	Achievement	Value of Education	Time Investment	Achievement
(1) value of education and self-rated performance	0.419 ***	0.566 ***	0.156 ***	0.344 ***	0.129 ***	0.407 ***			
(2) value of education and class rank	0.424 ***	0.541 ***	0.137 ***	0.395 ***	0.115 ***	0.506 ***			
(3) value of education, time investment, and self-rated performance							0.152 ***	0.390 ***	0.353 ***
(4) value of education, time investment, and class rank							0.151 ***	0.371 ***	0.386 ***
Cross-lagged effects (γ)	Value of education → achievement	Achievement → Value of education	Value of education → achievement	Achievement → Value of education	Value of education → achievement	Achievement → Value of education	Value of education → time investment	Time investment → achievement	Achievement → value of education
(1) value of education and self-rated performance	0.047 ***	0.197 ***	−0.014	0.077 ***	−0.006	0.062 ***			
(2) value of education and class rank	0.013 ***	0.546 ***	−0.010 ***	0.146 ***	−0.006	0.125 ***			
(3) value of education, time investment, and self-rated performance							1.818 ***	−0.002 ***	0.094 ***
(4) value of education, time investment, and class rank							1.703 ***	−0.001 ***	0.222 ***
Random effects and covariances	Value of education and achievement		Value of education and achievement		Value of education and achievement		Value of education and time investment	Time investment and achievement	Achievement and value of education
Intercept									
self-rated performance			0.141 ***		0.184 ***		3.569 ***	2.716 ***	0.137 ***
class rank			0.046 ***		0.065 ***		2.819 ***	1.187 ***	0.043 ***
Slope									
self-rated performance					0.002 ***				
class rank					0.001 **				
Intercept and slope									
self-rated performance					−0.023 ***				
class rank					−0.010 **				

Note: *** indicates that the *p* value of significant effects is lower than 0.001, ** indicates that the *p* value of significant effects is lower than 0.01.

The corrected Figure 8 and Figure 9 appear below.

**Figure 8:**
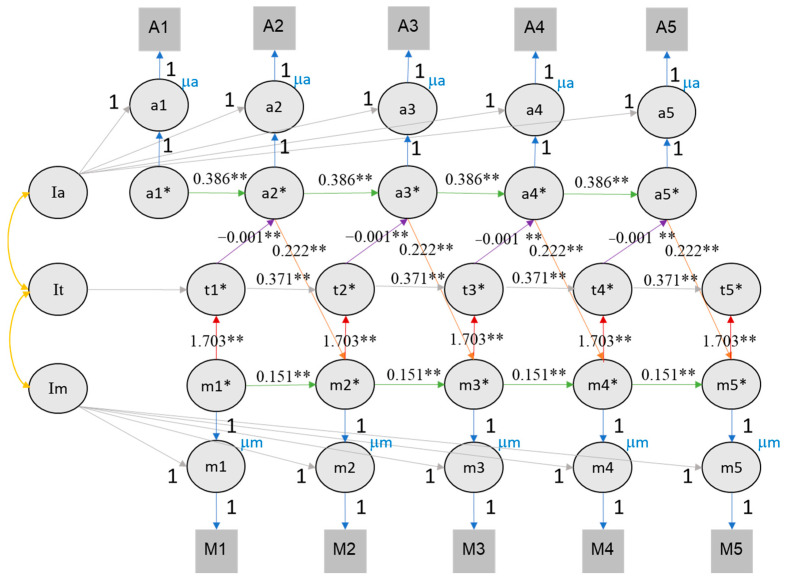
The RI-CLPM of value of education, time investment, and class rank. Note. Achievement here refers to self-rated performance in three subjects during the last semester. It is assumed that the achievement in the previous semester influences the motivation in the subsequent semester, which in turn affects the time investment in the current semester. The figures illustrate the reciprocal relationships among motivation (M/m), achievement (A/a), and time investment (t). Im, It, and Ia represent the intercepts of motivation, time investment, and achievement, respectively. Lowercase letters denote the time points from wave 1 to 5. Circles represent latent variables, while rectangles represent observed variables. The cross-lagged parameters are color coded (red, purple, and orange) to indicate different relationships, and autoregressive parameters are shown in green. The autoregressive and cross-lagged parameters are all equally constrained, hence the equal number of lines representing each parameter. The figures exclude the latent structure for error terms (e*) and associated variances and covariances for visual clarity. ** indicates that the *p* value of significant effects is lower than 0.01, * indicates that the *p* value of significant effects is lower than 0.05.

**Figure 9:**
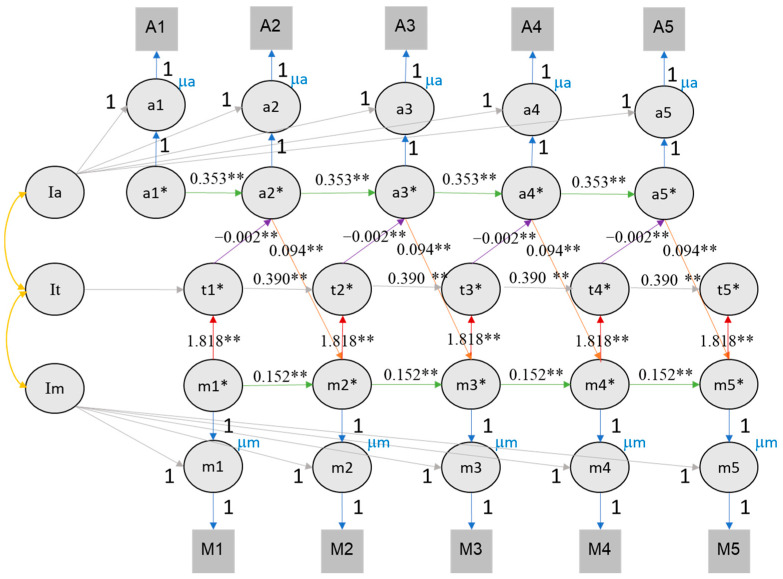
The RI-CLPM of value of education, time investment, and self-rated performance. Note. Conventions as in Figure 8. ** indicates that the *p* value of significant effects is lower than 0.01, * indicates that the *p* value of significant effects is lower than 0.05.

The following correction has been made to the relevant Section 4.5 of the Results section (see below). The corrected parts are highlighted with a bold font.

*4.5. Longitudinal Association of Value of Education, Time Investment and Academic Achievement*

To evaluate the role of time investment as mediating the effect between value of education and achievement, we expanded the RI-CLPM by including the time investment variable (see Figures 8 and 9). For this analysis, we only considered RI-CLPM and not RC-CLPM to limit complexity (given the many covariance parameters introduced in a trivariate RC-CLPM). Table 3 provides a comprehensive overview of all trivariate model parameters. The trivariate RI-CLPM model showed an acceptable fit for both self-rated **performance (CFI = 0.842, TLI = 0.833, RMSEA = 0.091, SRMR = 0.128) and class rank (CFI = 0.809, TLI = 0.797, RMSEA = 0.099, SRMR = 0.135)**. In the trivariate RI-CLPM, cross-lagged effects are represented by three links: value of education → time investment, time investment → achievement, achievement → value of education. Following the recommendations of Núñez-Regueiro et al. (2021), we report fit indices and significant effects for the fourth imputation for both rank and self-rated performance (see Supplementary Materials Section S2).

For class rank, a cross-lagged effect between value of education, time investment, and rank was found. The effect of value of education on time investment ranged from **1.729 to 2.137**, the effect of time investment on rank ranged from **−0.001 to 0.000**, and that of rank on value of education ranged **from 0.176 to 0.258** (see Figure 8). It is important to consider that the magnitude of these coefficients is influenced by the larger scale of time investment compared to other measures.

For self-rated performance, we found the same reciprocal effects between value of education, time investment, and achievement. From each time point to the next, the 95% confidence intervals of value of education to time investment effect ranged from **1.914 to 2.220**, the effect of time investment on self-rated performance ranged from **−0.001 to 0.000** (again affected by the larger scale of time investment), and the effect of self-rated performance scores on value of education ranged from **0.092 to 0.112** (see Figure 9).

The following correction has been made to the Discussion. The corrected parts are highlighted with a bold font.

Time investment → achievement: **Time investment was negatively, but weakly, correlated with achievement in the rank and self-assessed performance measures. This suggests that increased time investment does not lead to improved outcomes; in fact, it is negatively correlated with academic achievement. Similarly, (Marsh et al. 2016) examined the longitudinal reciprocal effects of academic self-concept, effort, and academic achievement among thousands of adolescents, and found that prior effort had non-significant or negative effects on subsequent grades. These results suggest that investing more time in learning may not always lead to higher achievement.**

The following correction has been made to the Abstract. The corrected parts are highlighted with a bold font.

**Abstract:** Many theories of motivation suggest that motivation and academic achievement reinforce each other over time, yet few longitudinal studies have examined behavioral pathways that may mediate interplay from motivation to achievement. Moreover, empirical studies so far have mostly focused on Western countries. In this study, we first examined whether students’ value of education, as a measure of motivation, is reciprocally related to achievement (class rank and self-rated performance) in a sample of junior high schoolers in an East-Asian country (N = 3445, Korean Youth Panel Study). We tested this reciprocity using different statistical models. Second, we investigated whether the relation between motivation and achievement was mediated by time invested in learning. Reciprocal effects between value of education and academic achievement were found in classic cross-lagged panel models, but only unilateral effects (from achievement to value of education) were found when we used random-intercept and random-curve cross-lagged panel models. **Adding the time investment variable, a reciprocal effect between value of education, time investment, and academic achievement was not found with the random-intercept model.** In conclusion, the reciprocity between of motivation and achievement are more elusive than previous research suggested; further studies should be dedicated to scrutinizing its existence with various statistical models.

The codes for constructing the tri-variate RI-CLPMs provided in the Supplementary Materials have been corrected on the OSF platform: https://github.com/MLi258/KYPS_codes.git, accessed on 1 June 2024.

In addition, the affiliation 2 in the original publication needs to be removed, and the affiliation numbers are changed accordingly.

The authors state that the scientific conclusions of the original article are unaffected. These corrections were approved by the Academic Editor. The original publication has also been updated.

## References

[B1-jintelligence-12-00117] Liu Meimei, Vu TuongVan, van Atteveldt Nienke, Meeter Martijn (2023). Testing the Reciprocal Effect between Value of Education, Time Investment, and Academic Achievement in a Large Non-Western Sample. Journal of Intelligence.

