# Supplementary material for: Testing the Reciprocal Effect between Value of Education, Time Investment, and Academic Achievement in a Large Non-Western Sample
Source: J Intell. 2023 Jul 4;11(7):133. doi: 10.3390/jintelligence11070133 (PMC10381180; doi:10.3390/jintelligence11070133)
Supplement: Supplementary file 1 [file jintelligence-11-00133-s001.zip › jintelligence-2320176-supplementary.pdf]

Supplementary Materials

Section S1: Factor analysis of motivational variables

This section will provide the factor analysis of 17 motivational items in the questionnaire. We conduct factor analysis in all five waves, found that 4 motivational items are included in one factor, which is our motivational variable: value of education.

Table S1. Codes of motivational questionnaire

q12a01w1	It is essential to get higher education for self-development
q1202w1	It is essential to get higher education in order to get a job
q1203w1	It is essential to get higher education in order to get ideal spouse
q1204w1	Getting higher education provides better opportunities for making good friends
q1205w1	People treat me better if I have higher education
q1206w1	Junior high school dropouts or people only with junior high school diploma receive serious discrimination in our society
q1207w1	I would like to get into social world earlier than others
q1208w1	I would like to earn money earlier than others
q1209w1	It is likely to earn more money with several part-time jobs rather than with one full-time job
q1210w1	I would like to become a freelancer since I do not want to be forced to follow rules and structure for firms and organizations
q1211w1	Junior high school dropouts or people only with junior high school diploma are very unlikely to get a job

q1212w1	I do not know much about my competencies and personal traits to make a career decision
q1213w1	I do not know my career options because I do not have enough information
q1214w1	Even though I know quite a lot about careers, I am attracted to a number of them, so it is difficult for me to choose one among them
q1215w1	I constantly make changes in my career plan
q1216w1	I find it difficult to make a career plan because of the differences between my parents and myself regarding my career plan
q1217w1	It is meaningless to set a career plan now because I do not know what the future will be like
a1218w1	In setting a career plan, I tend to follow my parents' recommendations more than my own opinion

Note. Each code corresponds to a question in the KYPS's questionnaire, and in this study we focus on four questions: q12a01w1, q12a02w1, q12a03w1 and q12a04w1. This four motivational question are included in one factor in all waves, below is the results:

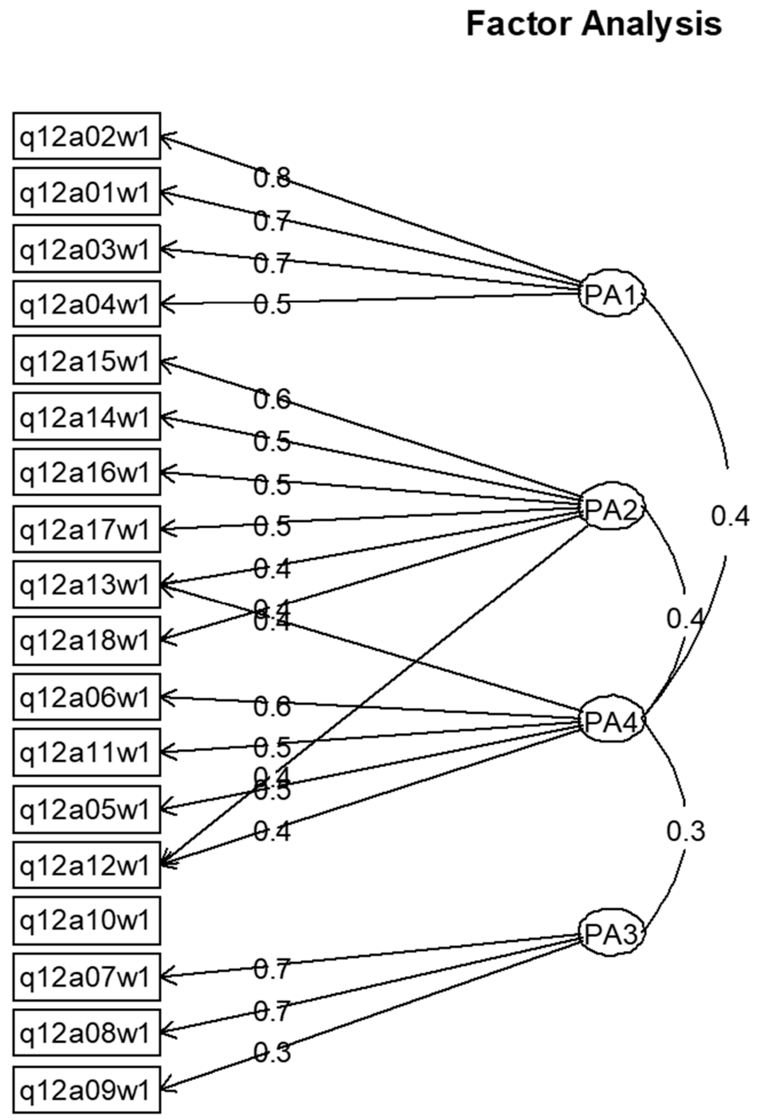

Figure S1. The factor analysis of 17 motivational items in wave 1.

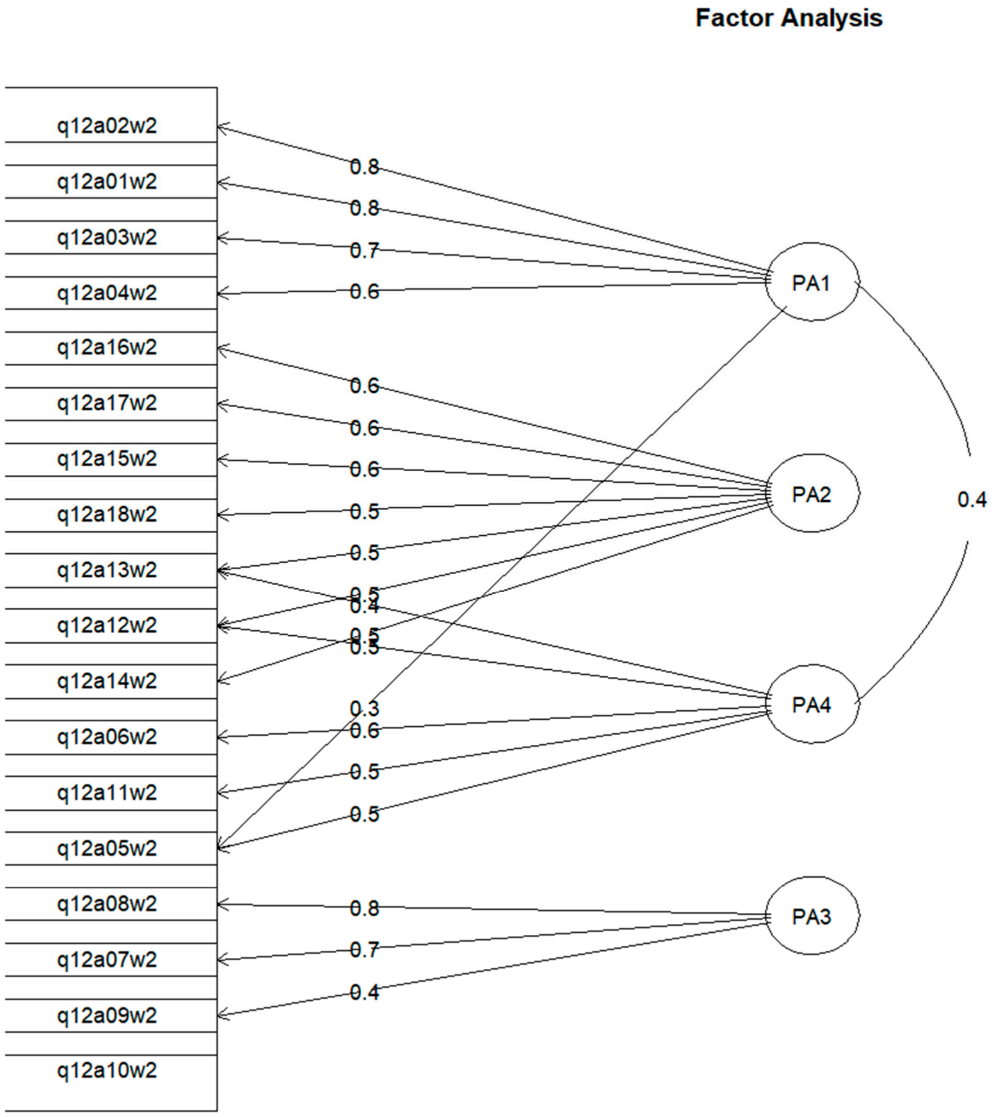

Figure S2. The factor analysis of 17 motivational items in wave 2.

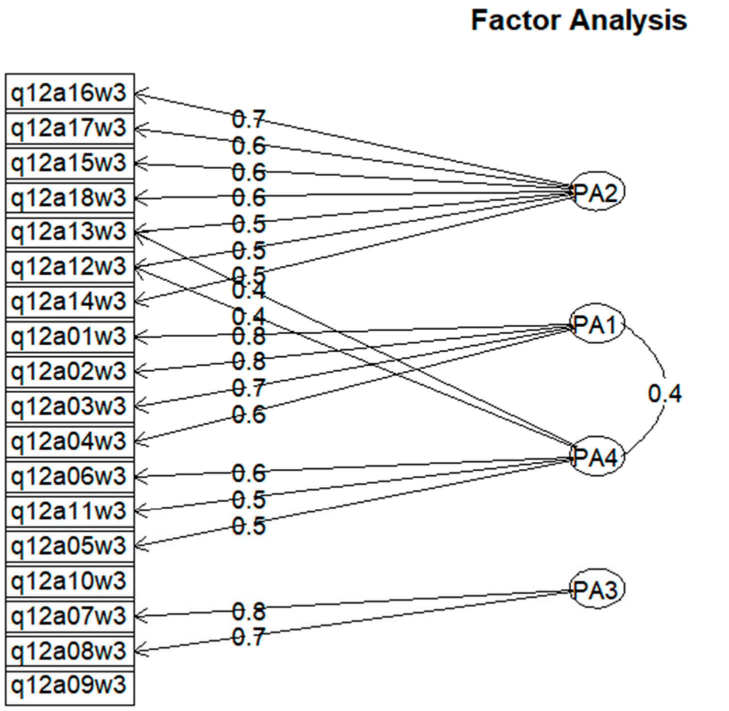

Figure S3. The factor analysis of 17 motivational items in wave 3.

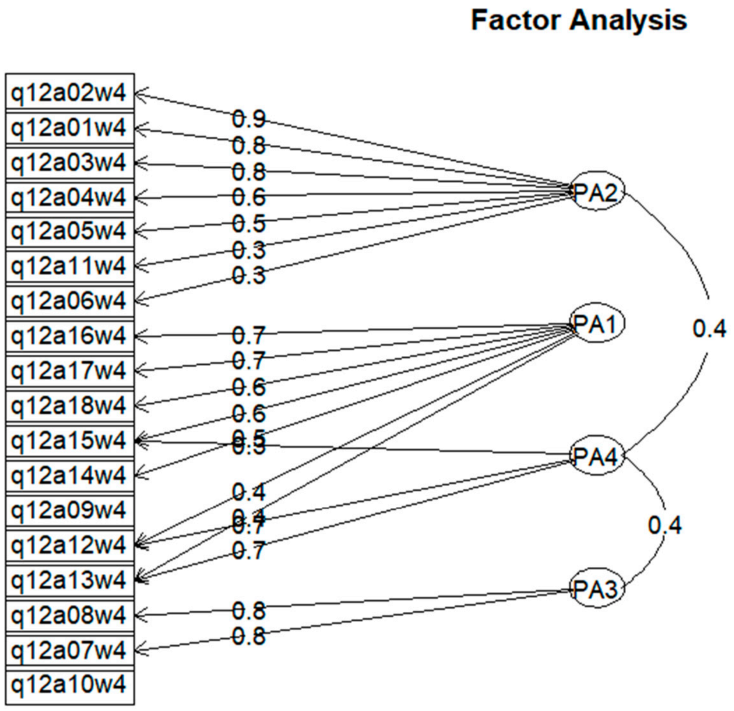

Figure S4. The factor analysis of 17 motivational items in wave 4.

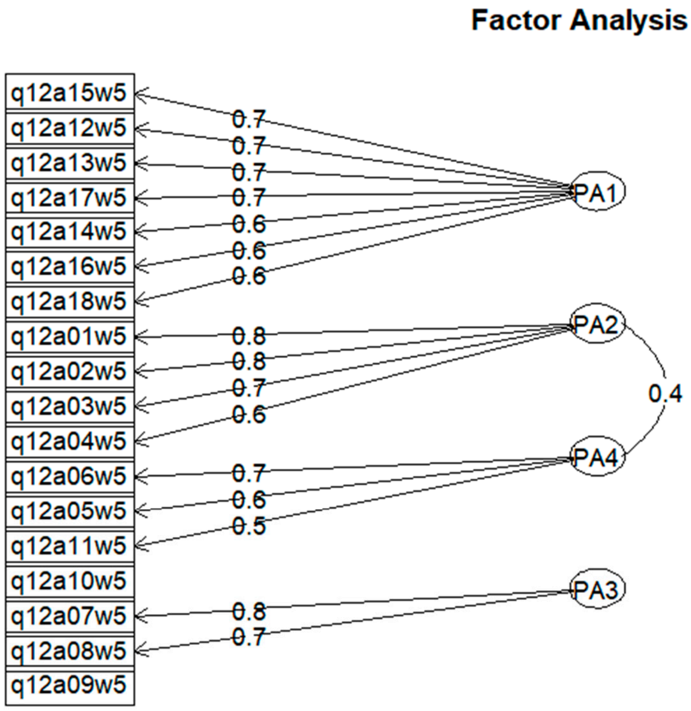

Figure S5. The factor analysis of 17 motivational items in wave 5.

Section S2: Multiple imputation results

This section will provide all multiple imputation results in 10 times in two achievement indicators (class rank and self-rated performance).

Table S2. 10 times imputation on CLPM in rank.

	DF	AIC	BIC	Chisq	Chisq diff	Df diff
fit1	46	37125	37242	2143.8		
fit2	46	37186	37303	2125.9	-17.804	0
fit3	46	37041	37158	2204.3	78.32	0
fit4	46	37080	37197	2133.5	-70.804	0
fit5	46	37193	37310	2108.8	-24.645	0
fit6	46	36994	37111	2172.3	63.47	0
fit7	46	36996	37113	2121.6	-50.662	0
fit8	46	37025	37142	2122.4	0.776	0
fit9	46	37190	37306	2141.6	19.194	0
fit10	46	37207	37323	2187.3	45.725	0

Note. Fit1-10 represents 10 times imputation under CLPM

Table S3. 10 times imputation on RI-CLPM in rank measures

	DF	AIC	BIC	Chisq	Chisq diff	Df diff
fit1	43	36277	36412	1288.8		
fit2	43	36322	36458	1256.2	-32.647	0
fit3	43	36148	36284	1305.5	49.385	0
fit4	43	36224	36359	1271.9	-33.609	0
fit5	43	36319	36454	1228.8	-43.137	0
fit6	43	36136	36271	1308.3	79.464	0
fit7	43	36164	36300	1284	-24.294	0
fit8	43	36151	36286	1241.5	-42.467	0
fit9	43	36329	36464	1275.1	33.638	0
fit10	43	36320	36455	1295	19.871	0

Note. Fit11-20 represents 10 times imputation under RI-CLPM

Table S4. 10 times imputation on RC-CLPM in rank measures

	DF	AIC	BIC	Chisq	Chisq diff	Df diff
fit21	40	35854	36008	860.64		
fit22	40	35919	36072	846.34	-14.297	0
fit23	40	35712	35865	862.98	16.638	0
fit24	40	35820	35974	861.87	-1.112	0
fit25	40	35886	36040	789.68	-72.186	0
fit26	40	35645	35799	811.73	22.052	0
fit27	40	35725	35879	838.74	27.006	0
fit28	40	35741	35895	826.06	-12.682	0
fit29	40	35875	36028	814.65	-11.412	0
fit30	40	35907	36061	876.08	61.439	0

Note. Fit21-30 represents 10 times imputation under RC-CLPM

Table S5. 10 times imputation on tri-variate RI-CLPM in rank measures

	DF	AIC	BIC	Chisq	Chisq diff	Df diff
fit31	94	177838	178090	2003.4		
fit32	94	178102	178354	1973.6	-29.849	0
fit33	94	178672	178924	2019.5	45.934	0
fit34	94	177374	177626	2014.3	-5.229	0
fit35	94	178274	178526	1948.5	-65.805	0
fit36	94	177697	177949	2056.9	108.451	0
fit37	94	178014	178266	1998.1	-58.865	0
fit38	94	177955	178207	1969.4	-28.62	0
fit39	94	178393	178645	1932.8	-36.68	0
fit40	94	178476	178728	2041.8	109.075	0

Note. Fit31-40 represents 10 times imputation under tri-variate RI-CLPM

Table S6. 10 times imputation on CLPM in self-rated performance measures.

	DF	AIC	BIC	Chisq	Chisq diff	Df diff
fit1	46	73584	73701	2060		
fit2	46	73510	73626	2020.6	-39.416	0
fit3	46	73534	73651	2043.5	22.89	0
fit4	46	73307	73423	1969.6	-73.911	0
fit5	46	73362	73478	2011.1	41.545	0
fit6	46	73410	73526	2075.4	64.271	0
fit7	46	73426	73543	2033.7	-41.702	0
fit8	46	73446	73563	2162.2	128.512	0
fit9	46	73556	73673	2104	-58.19	0
fit10	46	73473	73589	2054.8	-49.188	0

Note. Fit1-10 represents 10 times imputation under CLPM.

Table S7. 10 times imputation on RI-CLPM in self-rated performance measures.

	DF	AIC	BIC	Chisq	Chisq diff	Df diff
fit11	43	72558	72693	1027.71		
fit12	43	72509	72644	1013.55	-14.162	0
fit13	43	72518	72653	1021.53	7.985	0
fit14	43	72331	72466	987.55	-33.984	0
fit15	43	72376	72512	1019.86	32.308	0
fit16	43	72381	72516	1040.91	21.058	0
fit17	43	72415	72550	1015.99	-24.928	0
fit18	43	72388	72523	1098.28	82.29	0
fit19	43	72489	72624	1031.28	-66.995	0
fit20	43	72459	72594	1035.34	4.058	0

Note. Fit11-20 represents 10 times imputation under RI-CLPM.

Table S8. 10 times imputation on RC-CLPM in self-rated performance measures.

	DF	AIC	BIC	Chisq	Chisq diff	Df diff
fit21	40	72317	72470	780.74		
fit22	40	72297	72451	795.8	15.057	0
fit23	40	72268	72422	765.34	-30.46	0
fit24	40	72134	72288	784.8	19.457	0
fit25	40	72149	72303	786.46	1.665	0
fit26	40	72135	72288	788.69	2.226	0
fit27	40	72206	72360	801.77	13.083	0
fit28	40	72143	72297	847.8	46.029	0
fit29	40	72258	72412	794.16	-53.639	0
fit30	40	72259	72413	829.24	35.074	0

Note. Fit11-20 represents 10 times imputation under RC-CLPM.

Table S9. 10 times imputation on trivariate RI-CLPM in self-rated performance measures.

	DF	AIC	BIC	Chisq	Chisq diff	Df diff
fit31	94	214118	214370	1609.9		
fit32	94	214310	214562	1611.9	2.042	0
fit33	94	215060	215312	1622.9	11.009	0
fit34	94	213503	213755	1599.5	-23.417	0
fit35	94	214338	214590	1623.5	24.008	0
fit36	94	213981	214233	1687.8	64.28	0
fit37	94	214282	214534	1605.6	-82.17	0
fit38	94	214230	214482	1724.1	118.452	0
fit39	94	214584	214836	1597.7	-126.398	0
fit40	94	214606	214858	1619.7	21.994	0

Note. Fit21-30 represents 10 times imputation under on trivariate RI-CLPM.

Table S10. The distribution of missing data

	Motivation (4 items)	Time investment (12 items)	Class rank (1 item)	Self-rated performance (3 items)
Wave 1	4.03%	0.26%	0.63%	0
Wave 2	10.03%	7.57%	5.5%	7.68%
Wave 3	10.98%	9.39%	7.57%	10.61%
Wave 4	10.41%	9.51%	9.68%	12.32%
Wave 5	14.99%	14.24%	15.97%	17.51%

Note. If one construct included more than one item, then we reported the percentage of missing items for more than 2 items.

Table S11: The correlation between the average self-rated scores of five subjects, three subjects and class rank.

	X5a1	X5a2	X5a3	X5a4	X5a5	aw1	aw2	aw3	aw4	aw5	r1	r2	r3	r4	r5
X5a1	1														
X5a2	0.74	1													
X5a3	0.43	0.46	1												
X5a4	0.33	0.36	0.49	1											
X5a5	0.38	0.38	0.43	0.5	1										
aw1	0.93	0.69	0.39	0.31	0.35	1									
aw2	0.72	0.93	0.4	0.32	0.35	0.74	1								
aw3	0.45	0.46	0.89	0.49	0.42	0.45	0.46	1							
aw4	0.4	0.41	0.56	0.83	0.5	0.4	0.41	0.6	1						
aw5	0.44	0.45	0.53	0.55	0.83	0.43	0.44	0.56	0.65	1					
r1	-0.76	-0.7	-0.35	-0.28	-0.34	-0.72	-0.68	-0.37	-0.35	-0.38	1				
r2	-0.72	-0.73	-0.37	-0.3	-0.36	-0.69	-0.7	-0.38	-0.37	-0.41	0.83	1			
r3	-0.39	-0.4	-0.63	-0.49	-0.42	-0.35	-0.34	-0.61	-0.53	-0.51	0.39	0.41	1		
r4	-0.33	-0.34	-0.5	-0.57	-0.44	-0.29	-0.3	-0.49	-0.62	-0.54	0.34	0.36	0.67	1	
r5	-0.34	-0.33	-0.5	-0.51	-0.51	-0.32	-0.3	-0.5	-0.56	-0.65	0.36	0.39	0.64	0.73	1

Note. x5a1-x5a5 refers to the average self-rated scores of five subjects across five waves. aw1-aw5 refer to the average self-rated scores of three subjects across five waves. aw1-aw5 refer to the average self-rated scores of three subjects across the five waves. r1-r5 refers to the class rank across the five waves.

Table S12. Parameter estimates from different models using data with complete cases.

parameters	CLPM		RI-CLPM		RC-CLPM		3 variable RI-CLPM		
Autoregressive effects (β)	Value of education	achievement	Value of education	achievement	Value of education	achievement	Value of education	Time investment	achievement
(1)value of education and self-rated performance	0.427***	0.563***	0.153***	0.302***	0.135***	0.323***			
(2)value of education and class rank	0.424***	0.647***	0.156***	0.506***	0.139***	0.655***			
(3)value of education, time investment and self-rated performance							0.168***	1.292***	0.356***
(4)value of education, time investment and class rank							0.175***	1.359***	0.628***
Cross-lagged effects (γ)	Value of education → achievement	Achievement →Value of education	Value of education → achievement	Achievement →Value of education	Value of education → achievement	Achievement →Value of education	Value of education → time investment	Time investment → achievement	achievement →value of education
(1)value of education and self-rated performance	0.049***	0.214***	-0.002	0.130***	0.002	0.095***			

(2)value of education and class rank	0.010***	0.568***	-0.007***	0.183***	0.001	0.167*			
(3)value of education, time investment and self-rated performance							1.717***	0.008***	0.171***
(4)value of education, time investment and class rank							1.325***	0.003***	0.396***
Random effects and covariances	Value of education and achievement		Value of education and achievement		Value of education and achievement		Value of education and time investment	Time investment and achievement	Achievement and value of education
Intercept									
self-rated performance			0.130***		0.162***		1.717***	1,763***	0.095***
class rank			0.044***		0.061***		1.498***	0.005	0.021***
Slope									
self-rated performance					0.004***				
class rank					0.001**				
Intercept and slope									
self-rated performance					-0.022***				
class rank					-0.009***				

Note. The complete dataset includes 1421 participants.

Section S3: codes of modelling

This section provides R script for constructing three bivariate models and one trivariate RI-CLPM. Due to the length of codes, we upload it on github, which can be found in the website : KYPS_codes: This codes is for "Testing the reciprocal effect between value of education, time investment, and academic achievement in a large non-Western sample", we provide the codes for multiple imputation and model comparasion (github.com)
